# Developing the Society of Obstetric Medicine Australia and New Zealand (SOMANZ) Hypertension in Pregnancy Guideline 2023: Guideline development approaches, challenges and contextual considerations

**DOI:** 10.1002/gin2.70000

**Published:** 2024-09-16

**Authors:** Renuka Shanmugalingam, Zachary Munn, Timothy H. Barker, Helen L. Barrett, Amanda Beech, Lucy Bowyer, Tim Crozier, Amanda Davidson, Marloes Dekker Nitert, Aunty Kerrie Doyle, Luke Grzeskowiak, Nicole Hall, Hicham Cheikh Hassan, Annemarie Hennessy, Amanda Henry, David Langsford, Vincent W. Lee, Michael Peek, Joanne M. Said, Helen Tanner, Rachel Taylor, Meredith Ward, Jason Waugh, Linda Yen, Ellie Medcalf, Katy Bell, Deonna Ackermann, Robin Turner, Angela Makris

**Affiliations:** ^1^ Department of Renal Medicine Liverpool Hospital Liverpool New South Wales Australia; ^2^ School of Medicine Western Sydney University Sydney New South Wales Australia; ^3^ School of Clinical Medicine University of New South Wales Sydney New South Wales Australia; ^4^ Health Evidence Synthesis, Recommendations and Impact (HESRI), School of Public Health, Faculty of Health and Medical Sciences University of Adelaide Adelaide South Australia Australia; ^5^ Department of Obstetric Medicine Royal Hospital for Women Randwick New South Wales Australia; ^6^ Department of Obstetrics and Gynaecology Royal Hospital for Women Randwick New South Wales Australia; ^7^ Department of Intensive Care Monash Medical Centre Clayton Victoria Australia; ^8^ Monash Medical Centre Monash University Melbourne Victoria Australia; ^9^ Australian Pregnancy Hypertension Foundation Limited Surry Hills New South Wales Australia; ^10^ School of Chemistry and Molecular Biosciences University of Queensland Brisbane Queensland Australia; ^11^ College of Medicine and Public Health Flinders University Bedford Park South Australia Australia; ^12^ Department of Obstetrics and Gynaecology Liverpool Hospital Liverpool New South Wales Australia; ^13^ Department of Renal Medicine Wollongong Hospital Wollongong New South Wales Australia; ^14^ School of Medicine University of Wollongong Wollongong New South Wales Australia; ^15^ Department of Women's and Children's Health St George Hospital Sydney New South Wales Australia; ^16^ Department of Internal Medicine Grampians Health Ballarat Victoria Australia; ^17^ School of Medicine University of Melbourne Melbourne Victoria Australia; ^18^ Department of Renal Medicine Westmead Hospital Westmead New South Wales Australia; ^19^ Westmead Applied Research Centre, Faculty of Medicine and Health The University of Sydney Westmead New South Wales Australia; ^20^ School of Public Health, Centre for Kidney Research The University of Sydney Sydney New South Wales Australia; ^21^ Department of Maternal Fetal Medicine Centenary Hospital for Women and Children Canberra Australian Capital Territory Australia; ^22^ School of Medicine Australian National University Canberra Australian Capital Territory Australia; ^23^ Department of Maternal Fetal Medicine, Joan Kirner Women's and Children's Sunshine Hospital St Albans Victoria Australia; ^24^ Department of Obstetric Medicine Royal Brisbane and Women's Hospital Herston Queensland Australia; ^25^ Waikato Women's Hospital Hamilton New Zealand; ^26^ Department of Newborn Care Royal Hospital for Women Randwick New South Wales Australia; ^27^ Department of Obstetrics and Gynaecology, Faculty of Medicine and Health Sciences University of Auckland Auckland New Zealand; ^28^ Department of Rheumatology Te Whatu ora Counties Manukau New Zealand; ^29^ Sydney School of Public Health University of Sydney Sydney New South Wales Australia; ^30^ Biostatistics Centre University of Otago Dunedin New Zealand

**Keywords:** guideline challenges, guideline development, guideline methodology, hypertension in pregnancy

## Abstract

The Society of Obstetric Medicine Australia and New Zealand (SOMANZ) Hypertension in Pregnancy Guideline 2023 is the first evidence‐based guideline for hypertensive disorders of pregnancy that has been developed to the standards of the Australian National Health and Medical Research Council (NHMRC). This article describes the methodology, challenges and considerations in developing the guideline and includes details on group composition, literature search, evidence synthesis and development of recommendations. We also discuss several practical considerations and challenges related to contextual issues, topics of interest and resources available for guideline developers working with similar jurisdictions or topics.

## Highlights

- Discussion on the methodology used in developing a guideline to the standards of the Australian National Health and Medical Research Council (NHMRC).
- Discussion on the practical considerations and challenges faced by guideline developers in Australia and New Zealand.

## 1 INTRODUCTION

Hypertensive disorders of pregnancy (HDP) refer to a spectrum of hypertensive conditions in pregnancy that include pre‐eclampsia, chronic hypertension, gestational hypertension and superimposed pre‐eclampsia.^1^ HDPs affect approximately 3%–10% of all pregnancies annually and remains on the rise globally.^2^, ^3^, ^4^ In Australia and New Zealand, the incidence of pre‐eclampsia is reported to range from 3% to 4% of all pregnancies annually and is associated with significant maternal and foetal morbidity and mortality.^5^, ^6^, ^7^, ^8^ Several guidelines and position statements, including the Society of Obstetric Medicine Australia and New Zealand (SOMANZ) Guideline for the Management of Hypertensive Disorders of Pregnancy 2014, have provided guidance on the clinical management of HDP.^9^, ^10^, ^11^, ^12^, ^13^

This guideline is an update of the SOMANZ Guideline for the Management of Hypertensive Disorders of Pregnancy and is the first evidence‐based guideline for HDP developed to the standards of the Australian National Health and Medical Research Council (NHMRC).^14^

This article describes the methodology used in developing the guideline and includes details on group composition, literature search, evidence synthesis and development of recommendations. We also discuss several practical considerations and challenges related to contextual issues, topics of interest and resources available for guideline developers working with similar jurisdictions or topics. The full guideline can be accessed online at https://www.somanz.org/hypertension-in-pregnancy-guideline-2023/.^15^

## 2 OVERVIEW OF GUIDELINE METHODS

**Figure 1:**
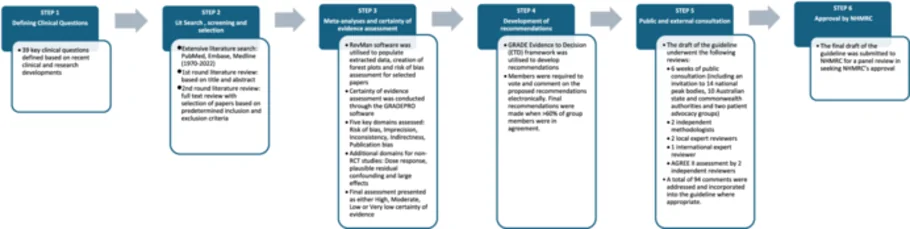
Summary of the methodology used in developing the SOMANZ Hypertension in Pregnancy 2023 Guideline.

The methodology for the guideline was developed in accordance with NHMRC's Guideline for Guidelines and followed GRADE methods (Figure 1).^14^, ^16^, ^17^ The guideline protocol was approved by the NHMRC Guideline Approval Programme in December 2020.^18^

### 2.1 Appointment of working group members

In 2019, SOMANZ identified the need to update the existing version of the society's HDP guidelines.^9^ The SOMANZ Executive Committee identified the guideline chair (A.M.), and an expression of interest for the lead of the group was advertised to the SOMANZ membership. The lead of the group (R.S.) was then appointed through a competitive process. The rest of the group members were identified based on their specific expertise and included a multidisciplinary team of clinicians, midwives, scientists, pharmacists, methodologists, First Nation People and consumer representatives from various states and territories across Australia and New Zealand. A guideline methodologist was appointed to assist with methodological decisions and a specialised team with epidemiological and biostatistical expertise was sought to assist with diagnostic test accuracy (DTA) assessments. Most of the working group members were unpaid volunteers who provided their time and expertise at no cost.

### 2.2 Defining the scope of the guidelines and identification of clinical questions

The working group determined the scope of the guideline and identified 39 critical clinical questions based on clinical and research developments since the last version of the guideline (Table 1).^9^ The final list of clinical questions was determined collectively based on current clinical priorities, cost of guideline development and access to resources. Where there was disagreement, an electronic voting poll was utilised to determine majority agreement. All clinical questions were framed using the PICO (Population, Intervention, Comparator and Outcomes) framework.^19^

**Table 1:** Critical clinical questions addressed in the guideline.

Theme	Clinical question
Screening	Utility of combined first‐trimester screening in identifying women at risk of pre‐eclampsia
Prevention	Use of aspirin in preventing pre‐eclampsia in women who are at high risk of developing pre‐eclampsia
Prevention	Use of calcium in preventing pre‐eclampsia in women who are at high risk of developing pre‐eclampsia
Prevention	Use of omega‐3 LCPUFA in preventing pre‐eclampsia in women who are at high risk of developing pre‐eclampsia
Prevention	Use of supplemental garlic in preventing pre‐eclampsia in women who are at high risk of developing pre‐eclampsia
Prevention	Use of antioxidants (vitamin C and E) in preventing pre‐eclampsia in women who are at high risk of developing pre‐eclampsia
Prevention	Use of oral magnesium in preventing pre‐eclampsia in women who are at high risk of developing pre‐eclampsia
Prevention	Use of progesterone in preventing pre‐eclampsia in women who are at high risk of developing pre‐eclampsia
Prevention	Use of low molecular weight heparin in preventing pre‐eclampsia in women who are at high risk of developing pre‐eclampsia
Prevention	Use of statin in preventing pre‐eclampsia in women who are at high risk of developing pre‐eclampsia
Prevention	Use of nitric oxide in preventing pre‐eclampsia in women who are at high risk of developing pre‐eclampsia
Prevention	Use of metformin in preventing pre‐eclampsia in women who are at high risk of developing pre‐eclampsia
Prevention	Use of oral vitamin D in preventing pre‐eclampsia in women who are at high risk of developing pre‐eclampsia
Prevention	Use of proton pump inhibitors (PPIs) in preventing pre‐eclampsia in women who are at high risk of developing pre‐eclampsia
Prevention	Use of clopidogrel in preventing pre‐eclampsia in women who are at high risk of developing pre‐eclampsia
Prevention	Increase in physical activity in preventing pre‐eclampsia in women who are at high risk of developing pre‐eclampsia
Prevention	Dietary salt restriction in preventing pre‐eclampsia in women who are at high risk of developing pre‐eclampsia
Diagnostic	Urine assessment for proteinuria in diagnosing pre‐eclampsia (utility of urine dipstick, urine protein to creatinine ratio and urine albumin to creatinine ratio)
Diagnostic	Clinical utility of the angiogenic biomarker, s‐Flt1/PlGF ratio, in ruling out and diagnosing pre‐eclampsia
Diagnostic	Clinical utility of PlGF‐base biomarker in ruling out and diagnosing pre‐eclampsia
Management	Ideal blood pressure target in the management of non‐acute (nonsevere) blood pressure in pregnancy
Management	Utility of home BP monitoring in monitoring women with stable chronic or gestational hypertension
Management	Choice of antihypertensives in the management of stable (nonacute/severe) hypertension in pregnancy
Management	Appropriate timing of birth in women with chronic hypertension or gestational hypertension
Management	Utility of ambulatory blood pressure monitoring in pregnancy
Management	Management of acute (severe) hypertension (≥160/110 mmHg) in pre‐eclampsia (appropriate treatment target and antihypertensives)
Management	Appropriate timing of birth in women with pre‐eclampsia
Management	Indication for corticosteroid in women with pre‐eclampsia at risk of preterm birth
Management	Indication for use of magnesium for foetal neuroprotection in women with pre‐eclampsia at risk of preterm birth
Management	Use of magnesium sulphate in minimising the risk of eclampsia and treating eclampsia
Management	Use of anticonvulsants in minimising the risk of eclampsia
Management	Use of corticosteroids in the management of HELLP syndrome
Management	Indication and use of venous thromboprophylaxis in women with pre‐eclampsia
Management	Use of plasma expansion in managing pre‐eclampsia
Immediate postpartum care	Use of nonsteroid anti‐inflammatories for postpartum pain management in women with pre‐eclampsia
Immediate postpartum care	Use of loop diuretics in managing hypertension in the postpartum period in women with pre‐eclampsia
Immediate postpartum care	Choice of antihypertensive agents in managing hypertension in the postpartum period
Long‐term postpartum care	Appropriate long‐term follow‐up schedule, investigations and management of women with pre‐eclampsia

### 2.3 Literature search, screening and selection

Comprehensive literature searches for studies from 1970 to 2022, based on predetermined MeSH (Medical Subject Headings) keywords, were conducted through three main electronic databases (Medline, Cochrane Library and Embase). Studies of non‐English literature, nonpregnant subjects and animal and cell studies were excluded. Details on MeSH keywords and literature search output have been included in the technical report and can be made available on written request to the corresponding author.

The selection of studies was conducted through a two‐step review process. Titles and abstracts were independently assessed by the lead (R.S.) and chair (A.M.) to exclude studies that met the specified exclusion criteria. Any disagreements were resolved following a review by a third member of the working group. The full text of selected abstracts was reviewed independently by two group members. Meta‐analyses, systematic reviews and randomised controlled studies were given preference over cohort studies and case–control studies, except for DTA clinical questions, where cross‐sectional studies were analysed. Where there were more than 10 randomised controlled studies (RCTs), we excluded cohort studies, case–control studies and retrospective studies. Any disagreements were resolved following a review by a third member of the working group.

### 2.4 Meta‐analyses and certainty of evidence assessment

Data extraction from included studies was performed independently by two members of the working group. Inconsistencies with data extraction were resolved following a review by a third member of the working group. Extracted data were populated into ReviewManager (RevMan 5.3) (The Cochrane Collaboration, 2021) to generate meta‐analyses and forest plots analysis. Where Cochrane systematic reviews with similar PICOs were identified, the publicly available Cochrane RevMan folders were reviewed, adapted and updated for analysis.

Dichotomous outcomes were expressed as risk ratios (RRs) with 95% confidence intervals (CIs), while mean difference (MD) with 95% CI was used for continuous outcomes. Data were pooled using the Mantel–Haenszel random‐effects model for dichotomous outcomes with two or more studies, while fixed‐effects model was used for dichotomous outcomes with less than two studies.

The analyses of DTA data were conducted by epidemiologists and a biostatistician with expertise in DTA. Forest plots displaying paired estimates of clinical sensitivity and specificity were constructed using the forest function from the DTA plots package in R (version 4.2.0).^20^ Where meta‐analysis was appropriate, summary estimates of sensitivity and specificity were calculated from bivariate models using STATA18 (StataCorp LLC, USA).

Certainty of evidence for all outcomes within each clinical question was assessed using the GRADE approach^17^ (conducted through the GRADEPro software) (https://www.gradepro.org/). Certainty of evidence for RCTs was downgraded based on risk of bias (RoB) assessment, inconsistency (heterogeneity), indirectness, imprecision and risk of publication bias. Nonrandomised studies were assessed for suitability for an upgrade from low certainty evidence according to the specified GRADE criteria (dose–response, plausible residual confounding and large effects); however, none of the nonrandomised studies examined met the criteria for the upgrade. Based on the domains assessed, an overall certainty of evidence of either high, moderate, low or very low was generated for each outcome examined. Summary of finding (SoF) tables with the synthesis of data and overall quality of evidence were generated for outcomes examined.

### 2.5 Development of recommendations

The GRADE Evidence to Decision (ETD) framework, which includes an assessment of the clinical problem, desirable and undesirable effects of the intervention, quality of evidence, values, balance of effects, acceptability and feasibility of intervention, resources required and equity, was utilised to facilitate judgement on recommendations. ETD for each clinical question, along with the SoF tables, were distributed to all members of the working group electronically through the GRADEPRO software. Members were required to vote and comment on the proposed recommendations electronically. The outcomes of the votes were discussed and finalised through two videoconferences. Final recommendations were made when >60% of group members were in agreement.

Recommendations in the guideline are presented as either evidence‐based recommendations or practice points (Table 2). Evidence‐based recommendations are presented with the strength of the recommendation and the quality of the evidence (Table 3).

**Table 2:** Difference between evidence‐based recommendations and practice points.

Evidence‐based recommendation	Practice points
Meta‐analysis and quality of evidence analysis conducted	Inadequate data to conduct meta‐analysis (no meta‐analysis conducted)
Recommendation generated based on evidence meta‐analysis and quality of evidence analysis	Recommendation generated based on consensus statement (limited or no data)
Guidance is based on evidence and is clinically actionable	Guidance to be used at the discretion of the clinician

**Table 3:** Strengths and grade of recommendations used in the Grading of Recommendations Assessment, Development and Evaluation methodology.

	Implication
Strength of recommendation	
Level 1 (strong)	Most women should receive the recommended course of action
Level 2 (weak)	Different choices will be appropriate for different women. The decision on treatment options should be made through a shared informed decision‐making process with the women
Grade and quality of recommendation	
A (high)	The working group members have a lot of confidence that the true effect is similar to the estimated effect
B (moderate)	The working group members believe that the true effect is probably close to the estimated effect
C (low)	The working group members believe that the true effect might be markedly different from the estimated effect
D (very low)	The working group members believe that the true effect is probably markedly different from the estimated effect

### 2.6 Public consultation

The draft guideline was open for public consultation for 6 weeks. Notification of the public consultation was made through SOMANZ's electronic newsletter and social media account. Invitations for public consultation were also sent directly to 14 national peak bodies, 10 Australian state and commonwealth authorities and two patient advocacy groups.

As part of the public consultation, NHMRC coordinated reviews by two independent methodologists, two local expert reviewers and one international expert reviewer. AGREE II assessments were conducted independently by two invited reviewers. Based on these reviews, a total of 94 comments were addressed and incorporated into the guideline where appropriate. The outcome of the public consultation can be accessed through the technical report, which can be made available on written request to the corresponding author.

## 3 GUIDELINE CHALLENGES AND CONSIDERATIONS

### 3.1 Guideline approval by an independent body

In Australia, guidelines can be approved by the NHMRC if they meet the necessary standards and procedures in NHMRC's Guidelines for Guidelines.^14^ This is an extra step in the guideline development process and can introduce several challenges, which include extended timelines, specific methodological requirements and the need for extensive public consultation. Nevertheless, the rigorous process results in the NHMRC‐approved guideline having greater recognition and impact, which vastly outweighs the logistical challenges.

Following public consultation, the final manuscript, along with a technical report, administrative report, response to public consultation, dissemination plan and implementation plan, was submitted to the NHMRC council for review. The guideline was reviewed, discussed and endorsed by the council in December 2023. The development of this guideline took a total of 39 months due to the need to meet NHMRC's requirements and the COVID‐19‐related temporary disruption (discussed below).

### 3.2 Developing recommendations on topics with inadequate data

The working group prioritised the selection of RCTs over observational studies, cohort studies and retrospective studies for evaluation of interventions. However, RCTs on certain key clinical topics, such as the clinical application of biomarkers, combined first‐trimester screening and post‐partum care of women with HDP were not available within specified search criteria. Therefore, limited analyses were conducted based on prospective and cross‐sectional studies and this is reflected in the grade of the recommendations made. In the absence of adequate data, practice points on the long‐term postpartum care of women with HDP were generated based on expert consensus and current clinical practice. These, however, remain critical clinical questions that require rigorous evidence‐based guidance. The group acknowledges that there are emerging studies on these topics and that the current recommendations are subject to updates in the near future (discussed below).

### 3.3 Development of guideline with the use of GRADE methods

Although this guideline was ostensibly an update of the 2014 guideline, it was more akin to a complete redevelopment as the methods utilised were vastly different. The GRADE methods were endorsed by NHMRC 2016; however, at the time of publication, many Australian guidelines had not adopted these methods.^21^

For the purpose of this guideline development, a methodologist, who oversaw much of the methods development, was appointed. In addition, all members of the working group undertook a two‐part online workshop with the JBI Adelaide GRADE centre, which covered competency training on evidence synthesis, assessment of the certainty of evidence through the GRADE system and the use of the ETD Framework in developing recommendations. The workshops on the use of GRADE and ETD frameworks facilitated a smoother recommendation development process, which consequently, allowed for a transparent, rigorous and trustworthy guideline.

### 3.4 Developing recommendations on new screening and diagnostic tools and equity implications

At the time of publication of the guideline, combined first‐trimester screening, which incorporates the use of maternal characteristics, placental biomarkers, sonographic characteristics and angiogenic biomarkers (s‐Flt1/PlGF ratio and PlGF‐based testing), was not widely accessible across Australia and New Zealand. Therefore, women's access to these diagnostic tools varied based on geographic location, affordability and availability, raising considerations of equity when developing recommendations. The working group, therefore, generated balanced recommendations using the ETD framework, which incorporates feasibility, cost, equity and a summary of evidence in developing recommendations.

Combined first‐trimester screening and biomarkers, however, remain important clinical tools that may be more easily accessible and implementable in the near future. The working group will continue to assess the availability and access to these diagnostic tools at a national level and update the recommendations as appropriate.

### 3.5 Update of guideline

Whilst NHMRC and most guideline committees recommend a review and update of guidelines within 5 years of publication, the rapid pace of emerging new data and studies often necessitates an update much sooner. The group acknowledges that data around the use of angiogenic biomarkers are rapidly evolving, even at the time of publication of this article, with new data on PlGF‐alone testing in screening and diagnosing pre‐eclampsia.

The group anticipates the need to update recommendations on clinical questions with significant new data within 2 years of publication. This update will be limited to specific clinical questions only. This method of update (by chapter or clinical question) would ideally be achieved through a living approach.^22^, ^23^ However, a living guideline requires resources including software and a team of dedicated specialists (information specialists, methodologists and data analysts) to ensure regular timely updates. Unfortunately, access to the required resources is often limited by lack of sustained funding, which, in turn, limits guideline groups from making frequent up‐to‐date recommendations, as is the case with this guideline.

### 3.6 Developing a guideline that is applicable to a diverse and multicultural society

Australia and New Zealand's populations consist of a diverse mix of women from various cultural, socioeconomic, education and linguistic backgrounds. It is also known that women of non‐English‐speaking backgrounds and those from lower socioeconomic backgrounds have a higher risk of adverse maternal and foetal outcomes.^24^, ^25^ The working group acknowledges the importance of ensuring that the recommendations in the guideline are applicable and accessible to all women, irrespective of their background.

The guideline consists of six patient infographics that have been produced in English, Vietnamese, Arabic, Hindi, Māori, Mandarin and are culturally appropriate for First Nation Women of Australia and Aotearoa New Zealand. The infographics and patient summary sheets were generated in plain language with the use of a Hemingway editor and in collaboration with consumer and First Nation People representatives. The users of the guideline are advised to counsel women and empower them with the multilingual infographic and patient summary sheets provided. These infographics are available for download by the public free of charge.^15^

### 3.7 Development of a guideline that is relevant to a multidisciplinary group of health professionals

Women with HDP are often cared for by a multidisciplinary group of physicians, obstetricians, midwives, maternal foetal medicine specialists, intensivists, pharmacists, scientists and neonatologists. Therefore, the working group was formed to reflect a multidisciplinary group that represents the diverse healthcare professionals involved in the care of women with HDP. In ensuring adequate dissemination, the guideline has been endorsed and advertised by national peak bodies that represent various groups of health professionals. The guideline also includes clinician information sheets, such as a summarised dietary calcium intake calculator, a venous thromboembolism risk assessment tool and postpartum care checklist, which are applicable to all health professionals who care for women with HDP. The clinician information sheets are available for download free of charge.^15^

### 3.8 Dissemination and implementation

A published guideline would not serve its intended purpose of enabling evidence‐based practice of medicine without adequate and effective dissemination. Clinical translation and implementation of the guidance are pivotal in improving patient outcomes; however, medical education today is multifaceted with increasing technology‐based learning with different types of learning requirements.^26^ Therefore, dissemination of the guideline in the current era will need to cater to contemporary learning styles. Furthermore, the rate of generation of new literature and the volume of knowledge continues to increase over time, necessitating the need for effective time‐efficient and easily digestible education tools for time‐poor healthcare providers.

In addressing this, this guideline provides an Executive Summary sheet of the recommendations, a Top 10 Summary sheet for patients and clinicians, summary flowcharts and checklists for healthcare providers and the publication of a summary article.^27^ These tools were disseminated with the assistance of various multidisciplinary professional peak bodies, mainly through their endorsement of the guideline and dissemination to their membership through their social media and electronic platforms.

### 3.9 Pandemic‐related challenges

A number of challenges were encountered during the COVID‐19 pandemic in guideline development.^28^ The development of these guidelines was disrupted for most of 2021 due to the COVID‐19 pandemic. A majority of the working group members are clinicians who are directly involved with patient care. In 2021, at the height of the COVID‐19 outbreak in Australia and New Zealand, the working group temporarily ceased to work on the guideline due to the critical need to prioritise clinical responsibilities. The group reconvened in early 2022 to resume guideline development.

As the pandemic‐related restrictions between 2020 and 2022 did not allow for in‐person meetings, all working group meetings and workshops were held on virtual platforms. The use of GRADEPRO and SurveyMonkey allowed for the use of electronic voting platforms in discussing and finalising recommendations.

### 3.10 Funding

Although developing guidelines with the rigour required to attain NHMRC approval is ideal, the cost implications need to be considered. Where funding and manpower are less available, the completion of the relevant aspects of guideline development is likely to require more time. This, in turn, is likely to result in task fatigue and significant delays in developing the guideline.

The availability of funding for guideline development can allow for better collaboration with expert groups to outsource various aspects of the guideline development, such as literature searching and data extraction. This is likely to allow for better collaboration in developing updates in a timely manner with a living guideline.^29^ Importantly, access to funding and collaboration will encourage the development of more evidence‐based guidelines to better guide healthcare providers in enabling best practice medicine.

## 4 CONCLUSION

This review paper describes the methods, challenges and considerations encountered in developing evidence‐based HDP guidelines. This guideline represents the first Australian and New Zealand HDP guideline that has been developed to NHMRC's standards and following GRADE approaches. The strategies we have used in the development of these guidelines were chosen to ensure the applicability of this guideline to all women and their healthcare providers across Australia and New Zealand.

## AUTHOR CONTRIBUTIONS

*Conceptualisation and writing of the manuscript*: Renuka Shanmugalingam, Angela Makris, Timothy H. Barker and Zachary Munn. *Proofreading of draft manuscripts and final manuscript*: Renuka Shanmugalingam, Zachary Munn, Timothy H. Barker, Helen L. Barrett, Amanda Beech, Lucy Bowyer, Tim Crozier, Amanda Davidson, Marloes Dekker Nitert, Aunty Kerrie Doyle, Luke Grzeskowiak, Nicole Hall, Hicham Cheikh Hassan, Annemarie Hennessy, David Langsford, Vincent W. Lee, Michael Peek, Joanne M. Said, Helen Tanner, Rachel Taylor, Meredith Ward, Jason Waugh, Linda Yen, Ellie Medcalf, Katy Bell, Deonna Ackermann, Robin Turner and Angela Makris.

## CONFLICT OF INTEREST STATEMENT

The authors declare no conflict of interest.

## ETHICS STATEMENT

No ethics application was required of this manuscript.

## Data Availability

The data that support the findings of this study are available from the corresponding author upon reasonable request.
